# Erratum

**DOI:** 10.1155/1994/38651

**Published:** 1994


					ERRATUM
HPB Surgery, 1994, vol. 8(1), pp. 62-65.
Paper title"
Emergency Portacaval Shunts in Variceal Hemorrage:
A Critique of Critics
by
Harold 0 Conn
Discussion on: Abstractby Odoff,M.J., Orloff, M.S., Rabotti, M. and Girard, B. (1992) Isportal-
systemic shunt worthwhile in Child's Class C cirrhosis. Annals of Surgery, 216, 256-268.
The paper by Orloffet al was published in Annals ofSurgery (1992), volume 216, pp. 256-268.
This is correctly given in the abstract, In the paper discussion, in the first sentence, the article is
incorrectly referred to as having been published in the American Journal ofSurgery. This should
read Annals of Surgery.
The author and publishers sincerely apologise for this error.

				

